# Parental Perspectives and Infant Motor Development: An Integrated Ecological Model

**DOI:** 10.3390/children12060724

**Published:** 2025-05-31

**Authors:** Ran An, Klaus Libertus

**Affiliations:** Department of Psychology, University of Pittsburgh, Pittsburgh, PA 15260, USA; klaus.libertus@pitt.edu

**Keywords:** motor development, infant development, parental influence, parental beliefs, parental cognitions, ecological systems theory

## Abstract

Infant motor development has traditionally been studied through child-centered frameworks that often overlook the vital role parents play in shaping early outcomes. This paper provides a renewed ecological approach, foregrounding parental perspectives—knowledge, beliefs, attitudes, theories, and expectations—and examining how they directly and indirectly guide infants’ motor trajectories. Drawing on cross-cultural evidence, we illustrate how differences in parental priorities and caregiving behaviors can either accelerate or delay the emergence of crucial motor skills. We also highlight the reciprocal relationship between parent and child: while parental views shape caregiving practices, children’s developing abilities and behaviors can, in turn, alter their parents’ perspectives. Building on existing theories, including Bronfenbrenner’s ecological systems theory and dynamic systems theory, our integrated model situates the parent–child dyad within broader socioeconomic, cultural, and environmental contexts. This model shows the dynamic, ever-evolving interplay between parents and children and demonstrates the importance of aligning parental cognition with targeted interventions to optimize motor development. By examining how cultural norms, individual experiences, and contextual factors converge, this paper offers both a theoretical framework and practical implications for supporting infants’ growth. This paper will inform future research by encouraging parent-focused developmental studies and guiding practitioners to design culturally informed interventions in the field of motor development.

## 1. Introduction

Child development is a multifaceted process that involves a dynamic interplay between children and their environment. Among the many factors influencing this process, parents play a particularly profound role in shaping early learning [1]. Researchers have long emphasized the importance of maternal care during infancy, as the quality of this care can either facilitate or hinder a child’s development [2]. When infants enter the world, they are entirely dependent on their caregivers, in many cases, their parents. As a child grows, parents play multiple roles, including feeding, protecting, educating, disciplining, and bonding with their children [3]. Through daily interactions, parents shape their children’s development both directly and indirectly. At the same time, parents also develop a sense of efficacy in their caregiving roles and establish expectations for their children [4]. Therefore, a deeper examination of parents’ roles during infancy is crucial for understanding the complexities of early childhood development.

One significant and often overlooked component of parental influence is the role of parental perspectives. In this paper, we use the term parental perspectives as a comprehensive concept that integrates a range of cognitive and affective constructs—including knowledge, beliefs, attitudes, theories, and expectations. While each of these components has been explored in past literature, there is no single term that adequately captures their combined influence on parenting behavior, especially within dynamic and culturally diverse contexts. We intentionally chose perspectives to reflect the holistic nature of how parents view development, themselves, and their children. This broader term allows us to account for both the explicitly held beliefs and tacit assumptions, which collectively shape caregiving behaviors. These perspectives act as guiding principles, determining which activities are introduced, how feedback is given, and how much effort is invested in caregiving [5]. These beliefs are active and evolving, in response to a parent’s own experiences, cultural background, and direct interactions with their child. Ultimately, parental perspectives create a framework that guides behavior and helps shape a child’s developmental journey across multiple domains, including motor skills.

The objectives of this paper are to (1) review the theoretical and empirical literature on the role of parental perspectives in motor development, (2) identify gaps in the existing frameworks, and (3) propose a novel integrated ecological model that positions parents as dynamic agents within child development. We begin by examining the foundational and contemporary theories that address motor development and the role of parents. Drawing from this theoretical foundation, we then review the empirical studies on how parental perspectives, caregiving behaviors, and environmental contexts shape motor trajectories. Based on the insights and gaps identified in these areas, we introduce a new ecological model that places the parent–child dyad at the center of the model. Finally, we reflect on the implications of this model for future research and intervention efforts, emphasizing the need to recognize parents as active, contextually embedded agents in shaping early motor outcomes.

### 1.1. Parental Perspectives in Academic Domains

Research has shown that parental perspectives significantly influence children’s developmental trajectories. This phenomenon has been primarily studied in the context of academic achievement. For example, children whose parents value math tend to perform better in that subject [6,7]. The mechanisms through which parental beliefs shape children’s math skills are important to understand. One proposed mechanism is that parents’ beliefs about the importance of math influence the types of activities they engage in with their children. Studies suggest that parents who place a high value on math are more likely to engage in math-related activities, such as counting and sorting, with their preschool and school-age children, thereby promoting their children’s math development [8,9,10,11,12]. Conversely, parental anxiety about their own math abilities is negatively associated with their children’s math performance [13].

Similar patterns emerge in literacy development. Parents who hold strong beliefs about the importance of literacy are more likely to provide early literacy experiences [14] and engage in positive interactions during joint book reading activities [15]. Through these home literacy practices, parents can foster both reading skills and motivation to read in their children [16]. Children whose parents believe reading is a source of entertainment show more interest in reading [17]. More generally, parents’ academic expectations and school readiness-related beliefs contribute to the quantity and quality of parenting practices and, in turn, influence children’s school readiness [18]. Interestingly, even non-academic beliefs can influence early learning. For example, parents who emphasize the importance of play are more likely to support activities that foster creativity, problem-solving, and social–emotional skills [19,20]. Additionally, maternal self-efficacy beliefs have been associated with toddlers’ cognitive outcomes and affection towards their mothers [21].

Collectively, these studies show that parental perspectives, whether specific to academic subjects or more generally, can shape children’s development by influencing the behaviors and environments that support learning. While much of this research has focused on cognitive skills like math and reading, other domains of development—particularly motor development—have received far less attention.

### 1.2. Importance of Motor Development

Motor development is critical for children’s overall growth and lays the foundation for other domains, such as language, social, and cognitive development [22,23]. Children are intrinsically drawn to move their bodies and discover new motor possibilities. These skills open new opportunities for children to explore their surroundings and engage in more social interactions, which in turn influences their further development [24,25,26,27].

For instance, the development of reaching skills in early infancy allows children to grasp and manipulate objects, facilitating object exploration and problem-solving skills [28]. Later, the emergence of locomotion skills, such as walking, enables infants to travel greater distances, experience new visual perspectives, interact with objects in various ways, and communicate more with caregivers [29]. This interconnection among motor skills, environment, and other domains of development is often explained through the concept of ‘developmental cascades’, where advancements in one area can elicit positive responses from the environment that promote further development [30,31]. For example, as a child learns to crawl, their parents may praise and encourage them, which gradually facilitates language learning and helps them make meaningful connections with their surroundings. This, in turn, promotes overall development. Therefore, understanding motor development is essential, as it acts as the building blocks for infant development.

While all infants achieve motor milestones as they grow, caregivers’ support is essential in this process. They contribute by providing physical space, engaging in play, and creating safe yet challenging environments for their children [3]. Understanding how parental perspectives influence motor development can reveal insights into these dynamics. Unlike cognitive domains, motor development research has historically received less attention regarding parental perspectives, though it is equally vital [23]. To contextualize motor development within a broader developmental framework, it is necessary to review the theoretical perspectives that have guided the research in this area. The following section will evaluate these theories to highlight their contributions and gaps, ultimately building the foundation for a renewed model. While this model emphasizes the relational and environmental influences on motor development, we acknowledge that child development arises from a complex interplay between innate biological factors and contextual experiences. Genetic predispositions and neuromotor readiness also play a vital role in shaping early motor skills.

## 2. Review of Theoretical Models

The theories on motor development provide frameworks for understanding how infants acquire motor skills and interact with their environments. Four prominent theories provide insights into motor development: the maturational theory, Bronfenbrenner’s ecological systems theory, Vygotsky’s sociocultural theory, and dynamic systems theory. Each of these frameworks offers a unique lens.

### 2.1. Maturational Theory

Traditionally, motor development has been studied through a biological lens, as exemplified by the maturational perspectives put forward by Arnold Gesell and others in the early 1900s [32,33,34,35]. Maturational views have remained popular and found widespread adoption in therapeutic settings. A key principle of the maturational theories of motor development is that motor development follows a biologically predetermined sequence of maturation, and environmental factors are considered to play only a minor role. Accordingly, infants are expected to follow a strict developmental sequence based primarily on internal readiness, regardless of external stimuli [36]. While this theory provides a foundational understanding of the biological underpinnings of motor development, its biologically determined view overlooks the significant role of experiences guided by parental engagement [37]. For example, maturational theories fail to explain cross-cultural differences in infant motor skill attainment [38].

### 2.2. Dynamic Systems Theory

A second theory that has found widespread adoption in the motor development literature is the dynamic systems theory. Dynamic systems views address the shortcomings of maturational perspectives by shifting the focus from fixed developmental sequences to the complex interplay of biological, environmental, and task-related factors that shape motor development [39,40,41]. According to this theory, children’s behaviors are organized into dynamic patterns that exhibit both stability and flexibility. By emphasizing self-organization and the adaptive nature of motor skill acquisition, this theory provides a valuable point of view regarding the complexity of developmental changes. However, dynamic systems theory has been criticized for its vague conceptualization of systems and its lack of specificity in identifying the causes of developmental change. This broad focus can make it difficult to predict or identify specific causal mechanisms [42]. In this theory, systems are rather abstract entities that exert some influence on a child (also known as the developing system). However, little attention is paid to these systems themselves. Specifically, while dynamic systems views acknowledge that parents dynamically and reciprocally influence their child, the theory does not address the parent’s own behavior, goals, or beliefs–the dynamic systems theory does not try to explain why the systems act the way they do.

### 2.3. Sociocultural Theory

Although not exclusively focused on motor development, other broader theories, such as sociocultural theory and ecological systems theory, are also relevant. Lev Vygotsky’s sociocultural theory offers profound insights into how social interactions and cultural norms shape individual development [43]. A central concept of Vygotsky’s theory is the Zone of Proximal Development (ZPD), which highlights the difference between what a child can achieve independently and what he/she can achieve under the guidance of a more knowledgeable other, such as a caregiver, peer, or teacher. This process of scaffolding allows children to integrate new knowledge into their existing mental structures. Vygotsky’s framework emphasizes the importance of social and cultural contexts in learning. However, the theory may not be universally applicable, as different social groups may employ diverse learning methods, and each individual’s social experience is unique [44].

### 2.4. Ecological Systems Theory

While Vygotsky’s theory underlines social interactions and cultural contexts, Urie Bronfenbrenner’s ecological systems theory provides a more comprehensive view of the bidirectional influences of the environment on child development. The ecological systems theory [45,46,47] complements other theories by situating the child within a complex system of relationships across multiple environmental levels. These interconnected systems range from the immediate family and close friends (microsystem) to broader cultural and societal influences (macrosystem). The exosystem, mesosystem, and chronosystem provide additional layers that contribute to a child’s upbringing. Despite its wide application, ecological systems theory has faced some criticism. For instance, some scholars have argued that culture is deeply integrated into daily activities and should not be placed in a separate, distal system [48].

Despite the theoretical advancements, gaps remain in understanding the role of parental perspectives in infant motor skill development. The ecological systems theory and sociocultural theory may not fully apply to all social groups, as caregiving practices and interactions between parents and children can vary widely across cultures and communities. Therefore, the dynamics between parents and children can differ significantly, limiting the universality of these theories. Moreover, while these theories provide valuable insights, they are not specifically tailored to motor development during infancy. Factors, such as school and peers, may not be applicable.

In terms of the theories that do focus on motor development, the maturational theory neglects the influence of environmental factors, suggesting that development is driven primarily by internal biological processes. On the other hand, the dynamic systems theory acknowledges the role of environmental variables, but its vague conceptualization of systems and subsystems makes it difficult to apply to specific contexts. Most importantly, all of the previous theories position the child at the center of the developmental model. While this perspective is critical, it has failed to look into the essential role that parents play, especially during infancy.

### 2.5. Other Theories

Although our model is grounded in behavioral and ecological perspectives, we acknowledge that theories, such as Neuronal Group Selection Theory (NGST), offer a complementary neurobiological perspective. NGST proposes that motor development involves the selection of efficient neural networks through a process of variation and experience-driven adaptation [49,50]. Although NGST is more focused on internal neurodevelopmental processes, its emphasis on variability and experience aligns broadly with our model’s attention to contextual influences. While NGST offers an important framework for understanding motor variability at the neural systems level, it is beyond the scope of this paper.

Similarly, the Developmental Niche framework [51,52] has informed the structure of our ecological layer. This model highlights how a child’s development is shaped by three interrelated systems: the physical and social settings of the child, the culturally structured caregiving practices, and the caretaker’s psychology. While this framework offers a culturally grounded lens and emphasis on the child’s developmental context, including their parents, it does not focus specifically on observable child behaviors as developmental outcomes. Our model builds upon this framework by integrating its cultural and relational components into a system that centers the parent–child dyad and explicitly links parental perspectives, behaviors, and child motor development within an ecological structure.

## 3. Review of Empirical Studies

The empirical research provides valuable insights into the mechanisms through which parental perspectives and behaviors influence infant motor development. Prior research examining whether and how parental perspectives may influence child development can be categorized into explicit parental perspectives, implicit caregiving behaviors, and intervention-driven changes. Together, these studies illuminate the pathways by which parental perspectives shape behaviors, how these behaviors directly impact motor outcomes, and how contextual factors influence both perspectives and practices.

### 3.1. Explicit Assessments of Parental Perspectives on Motor Development

The research on explicit parental perspectives provides a window into how parents conceptualize motor development and the ways these conceptualizations shape their caregiving behaviors. Parental perspectives are influenced by cultural norms, socioeconomic conditions, and personal experiences, creating significant variability in the practices that ultimately affect infant motor outcomes. However, research that focuses on parental perspectives in the motor development domain remains scarce. The few studies that have focused on the role of parental perspectives have been completed mainly outside of the United States.

In Brazil, the research has shown that parental attitudes toward motor development can shape parenting practices and infant outcomes. For example, Gomes et al. [53] asked parents about their beliefs and practices. It was observed that parents exhibited different approaches based on their beliefs. Parents who valued motor stimulation were more likely to actively encourage motor stimulation, offering toys and spending time on the floor with their children to practice sitting and walking. Additionally, a more recent study by Graciosa et al. [54] demonstrated that parents who viewed motor development as a natural process tended to have children with later-emerging independent walking skills. Together, these two studies show that parental beliefs or practices may accelerate or slow down motor skill development.

In England, a series of studies by Hopkins & Westra [55,56] compared Jamaican mothers living in England with their English counterparts. The results of these studies revealed surprising contrasting patterns. The Jamaican mothers expected their children to achieve sitting and walking earlier than the English mothers. These expectations were quite accurate, as the Jamaican infants did reach these milestones earlier. Interestingly, 3 English infants progressed from sitting to walking without having crawled, while 12 Jamaican infants achieved walking alone without crawling. These observations may be related to differences in beliefs. The Jamaican mothers viewed sitting and walking as significant steps toward independence and success, whereas crawling was seen as primitive and hazardous. These views were not held by the British mothers, resulting in different motor development trajectories across the two groups.

In Isreal, infants have been observed to reach certain motor milestones earlier than their American peers, while Dutch infants tend to reach them later [57,58]. These developmental differences correspond with the distinct parental beliefs and caregiving strategies in each culture. Israeli parents tend to place significant importance on motor development and often engage in stimulating practices, such as prone positioning and floor play. In contrast, Dutch parents generally emphasize rest, regularity, and allowing development to unfold at a child’s own pace. These patterns have been confirmed by recent cross-cultural research, which identified two contrasting cultural models of parenting beliefs: one emphasizing active facilitation as a parent in Israel, and the other prioritizing autonomy and supportive caregiving in the Netherlands [59,60]. Together, these findings highlight how observed developmental outcomes are shaped by underlying parental belief systems and broader cultural values.

The research on several African communities provides additional context. In these communities, infants often achieve motor milestones significantly earlier than their American and European counterparts [61]. This advancement is often attributed to broader societal factors. In these communities, motor skills are often encouraged from an early age, with parents employing hands-on methods to promote skills like sitting and standing [62]. For example, Yoruba mothers have reported that the age at which babies attain motor milestones depends on how they are reared. Some mothers have even suggested that children will not achieve these milestones without active teaching. These beliefs are coupled with cultural traditions. Yoruba caregivers often prop infants into sitting positions and place them in cardboard boxes or basins, believing these practices help foster independent sitting [63].

The reviewed studies highlight the significant variability in parental beliefs and behaviors, not only across cultures but also within them, revealing the complexity of the relationship between caregiving practices and motor development. In Brazil, the research demonstrates that even within the same cultural context, parental attitudes toward motor development can vary widely. Some parents actively promote motor stimulation, engaging in practices, such as floor play and the use of toys, to encourage sitting and walking [53]. In contrast, others adopt a more hands-off approach, viewing motor development as a natural process that unfolds without the need for direct intervention [54]. These differing perspectives align with distinct caregiving strategies and are potentially associated with variations in the timing of motor milestones, such as the emergence of independent walking. Cultural contrasts across countries further emphasize the influence of parental values on caregiving practices. Jamaican mothers prioritize sitting and walking, connecting these milestones with independence and maturity, while Israeli parents emphasize prone positioning to encourage the development of early physical skills [55,56,57]. In contrast, Dutch parents adopt a self-paced developmental philosophy, linked with a slower attainment of motor milestones [58]. These studies collectively reveal the nuanced relationships among parental perspectives, caregiving behaviors, and motor outcomes, demonstrating variability both within and across cultural contexts. They raise important questions about the factors that influence these relationships and challenge assumptions of uniformity in caregiving practices and beliefs, even within a shared cultural framework.

### 3.2. Implicit Assessments of Parental Perspectives on Motor Development

While explicit assessments provide insights into the indirect influences of parental perspectives, implicit assessments reveal the direct connections between caregiving practices and motor development. These studies often examine how parenting behaviors, shaped by cultural values and societal norms, impact infants’ motor trajectories. Although these practices may not always reflect consciously articulated beliefs, they provide critical information about the parent–child dyad.

Research conducted in Kokwet highlighted how societal norms shape proactive caregiving. Super [64] observed that over 80% of mothers reported actively teaching their children skills, such as sitting and standing, demonstrating a deeply embedded cultural emphasis on fostering motor development. Similarly, in Zambia, parents engage in culturally specific techniques to encourage motor skills, such as tossing infants to increase their arousal and muscle tone or placing them in slings to promote postural stability [65,66]. These practices are associated with advanced skills, such as sitting and standing, but with delayed crawling compared to Euro-American infants, reflecting differing cultural values about which motor milestones are prioritized. In contrast, Euro-American parents view crawling as an essential milestone, linking it to early locomotor ability, autonomy, and environmental mastery [67]. To promote these abilities, they encourage independent locomotor efforts and reinforce quadrupedal movements [38].

The delayed development of certain skills due to caregiving practices is another theme in implicit assessments. For instance, in rural Chinese communities, parents often do not emphasize motor milestones and engage minimally in interactive play with their infants. Wang et al. [68] observed that this lack of emphasis is associated with delayed motor skills and other developmental domains, suggesting that parental behaviors significantly influence developmental outcomes. A more striking example comes from Tajikistan, where the use of the “gahvora”—a traditional cradle that binds infants for prolonged periods—limits movement and leads to delayed milestones, such as crawling and walking [69,70]. In a recent comparative study, Wang et al. [71] extended these findings by examining motor restriction and infant locomotion in three culturally distinct societies: Tajikistan, Aka, and Tanna. They found that infants in Tajikistan experienced the longest durations of physical restriction due to cradle use, while Aka and Tanna infants were given far more opportunities to move around their neighborhoods and explore. Altogether, these findings illustrate how implicit caregiving practices, informed by cultural traditions, can both nurture and constrain opportunities for movement.

Within-cultural variations in parenting behaviors provide additional insights. In the United States, the “Back to Sleep” campaign, which promoted supine sleeping to reduce sudden infant death syndrome (SIDS), led many parents to restrict the use of prone sleeping positions [72]. Davis et al. [73] found that this shift was associated with delayed motor milestones, such as rolling and crawling. However, infants who regularly participated in tummy time or were placed in prone positions during waking hours demonstrated significantly higher locomotion scores [74]. These findings suggest that while parental practices may not always explicitly prioritize certain motor activities, these implicit decisions have measurable effects on infants’ motor development.

Cultural priorities regarding motor milestones also highlight the variability in implicit caregiving practices. For example, in Euro-American families, where crawling is viewed as a critical developmental step, parents may unconsciously encourage behaviors that facilitate independent mobility. Conversely, in communities where early sitting or standing is emphasized, practices like tossing or propping infants reflect an implicit belief in the importance of fostering postural control and balance.

Altogether, these examples demonstrate that caregiving practices are shaped by cultural values, societal norms, and individual parenting strategies. They show how parental behaviors influence motor trajectories by creating opportunities or imposing limitations. While these behaviors may not directly reflect parents’ conscious perspectives, they provide a valuable lens for understanding how implicit priorities and beliefs drive caregiving decisions and, consequently, infant development.

### 3.3. Research on Intervention Programs for Parents Points to Changes in Parental Behaviors

Intervention programs provide critical insights into how parental perspectives and behaviors can be influenced to support infant motor development. These programs target either the explicit and implicit aspects of parenting by addressing parental beliefs, modifying caregiving practices through structured activities, or fostering new skills. By engaging parents as active participants, interventions highlight the mechanisms through which parent-focused approaches can foster both immediate and long-term developmental outcomes.

Programs designed to explicitly modify parental perspectives demonstrate how changes in beliefs can influence caregiving behaviors and child outcomes. The Newborn Individualized Developmental Care and Assessment Program (NIDCAP) exemplifies this approach. By educating parents about infant behavioral cues and caregiving techniques, the NIDCAP aims to reduce parental stress and enhance parent–infant interactions. Participants have reported improved confidence in interpreting their infant’s needs and greater adaptability in caregiving strategies, leading to better motor outcomes for their children [75]. Another program by Moxley-Haegert and Serbin [76] supports this notion. In this study, a home treatment program was implemented to help developmentally delayed children. The caregivers who received parent education were more knowledgeable and had a better understanding of their children’s development than the no-education control group. Their children also had better motor outcomes as well. Moreover, at the follow-up visit a year later, the caregivers who received education participated more in treatment programs and were more motivated than those in the control group. Their children also showed continued advancement in their motor skills. These changes seemed to be long-lasting, demonstrating the benefit of support and education for parents.

The implicit influences on parental perspectives that indirectly shape behaviors have been observed in both clinical and non-clinical settings. Clinical programs targeting infants at risk of developmental delays have demonstrated the importance of parental involvement for achieving positive outcomes. For instance, interventions emphasizing motor-enhancing exercises rely heavily on parents’ active participation, and studies have shown that greater parental involvement correlates with improved motor milestones [77,78]. These findings suggest that interventions not only improve infant outcomes but also enhance parents’ understanding of their role as facilitators of development. Importantly, parents involved in such programs have often reported lasting changes in their caregiving behaviors, even after the intervention concluded [79].

Non-clinical interventions illustrate the transformative potential of parent-led practices integrated into daily routines. For example, Lobo and Galloway [80] demonstrated that parents trained in enhanced handling and positioning techniques for three weeks observed significant improvements in their infants’ gross and fine motor skills. Similarly, “sticky mittens” interventions, where parents guide infants in enriched reaching sessions, resulted in increased manual exploration, better coordination, and stronger parent–infant engagement [81,82]. These interventions influence parental behaviors by embedding motor skill practices into naturalistic interactions, ensuring that the changes are practical and sustainable.

The mechanisms driving changes in parental perspectives and behaviors are multifaceted. Education programs directly influence explicit beliefs by equipping parents with knowledge about developmental processes and caregiving techniques. For instance, by learning the importance of tummy time or handling exercises, parents may adopt more structured motor-stimulating behaviors. Behavior-focused interventions rely on experiential learning, where parents see tangible improvements in their child’s development, reinforcing the value of their caregiving practices. These experiences create positive feedback loops, where successful engagement strengthens parental confidence and commitment to motor-focused caregiving.

Whether these changes last depend on several factors, including the intensity and frequency of the intervention, the support available to parents post-intervention, and how well the practices align with parents’ cultural and ecological contexts. Studies like those by Lobo and Galloway [80] have demonstrated that when interventions are integrated into daily routines and supported by follow-up resources, parents are more likely to sustain the changes over time.

Collectively, these examples illustrate that interventions targeting both parental perspectives and behaviors can influence caregiving practices and create lasting impacts on infant motor development. By providing parents with practical skills, reinforcing positive outcomes, and addressing cultural and contextual factors, these programs equip families with the tools necessary to sustain nurturing caregiving environments.

## 4. New Integrated Model

While the existing theories and empirical studies offer valuable insights into motor development, significant gaps remain in understanding how parental perspectives dynamically influence caregiving behaviors and motor outcomes within diverse ecological contexts. The existing theoretical models, such as Bronfenbrenner’s ecological systems theory and dynamic systems theory, recognize the importance of environmental and interactive factors, but they often overlook the active role of parents, particularly how their beliefs and behaviors dynamically influence motor outcomes. These frameworks lack an integration of parental perspectives as the central agents of developmental change, especially during infancy.

**Figure 1:**
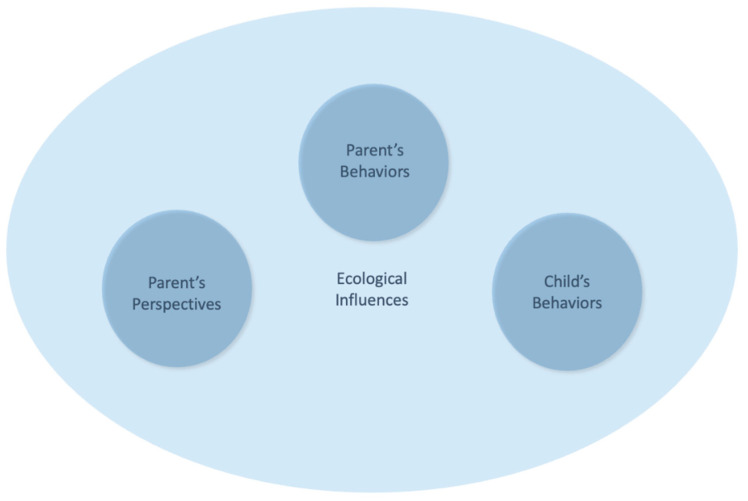
Integrated model of parent–child dyad in motor development. *Note*: This figure illustrates the new integrated model that places the parent–child dyad within an ecological framework, emphasizing the dynamic interplay among parental perspectives, parental behaviors, and children’s behaviors. It highlights the evolving nature of these interactions and demonstrates the importance of environmental contexts in shaping motor outcomes.

To address the existing gaps, we propose a new integrated model that situates the parent–child dyad within an ecological framework, emphasizing the dynamic interplay among parental perspectives, parental behaviors, and infant motor development (see Figure 1). By consolidating the existing theories, the model is structured around three core components: parental perspectives, parental behaviors, and children’s behaviors. These interactions are embedded within an ecological layer that represents the unique contexts of both parents and children. This structure is conceptually informed by the Developmental Niche framework [51,52], which identifies the physical and social settings of the child, culturally regulated caregiving, and caregiver psychology as the key organizing systems in development. Our model draws on these subsystems but departs from the Developmental Niche framework by placing a stronger emphasis on parental perspectives as active drivers, and by focusing explicitly on motor behavior as a developmental outcome.

The model recognizes that parental perspectives and behaviors evolve through interactions with their children and their broader environment. It also draws attention to the importance of cultural and socioeconomic contexts in shaping these perspectives and practices, providing a comprehensive understanding of how parents influence their children’s development. By focusing on the reciprocal nature of the parent–child dyad and the contextual factors that shape these interactions, this model offers new insights into the mechanisms through which parental perspectives affect motor outcomes.

### Model Components

**Figure 2:**
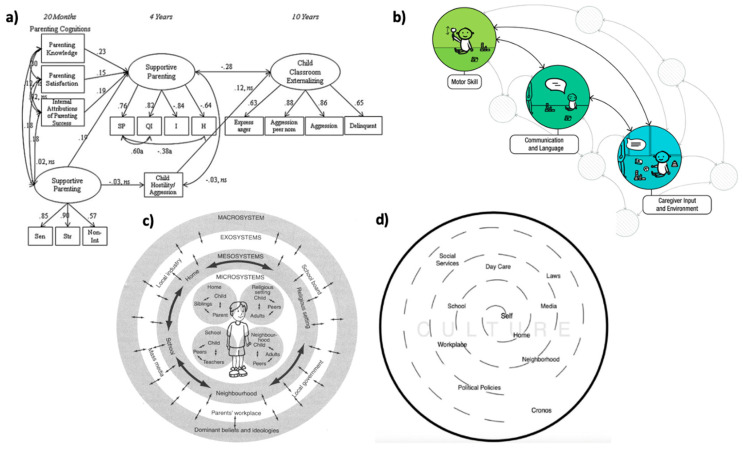
Illustrations informing the integrated framework. *Note*: This figure presents four illustrations that inspired the development of the proposed integrated framework. (**a**) is taken from Bornstein and colleagues [5], and demonstrates how parental cognition shapes parenting practices, which in turn influence child outcomes. (**b**) is taken from Iverson [30], and illustrates the developmental cascade model, where a child’s motor development influences their surroundings, including parental behaviors. (**c**) is taken from Bronfenbrenner’s ecological systems theory [45], and situates a child within a series of systems, from immediate family to broader societal norms, all of which impact the child’s development bidirectionally. Finally, (**d**) is taken from Vélez-Agosto and colleagues [48], and includes the revised ecological model, which moves cultural factors into the microsystem. These models together serve as the theoretical foundation for the proposed model.

**Figure 3:**
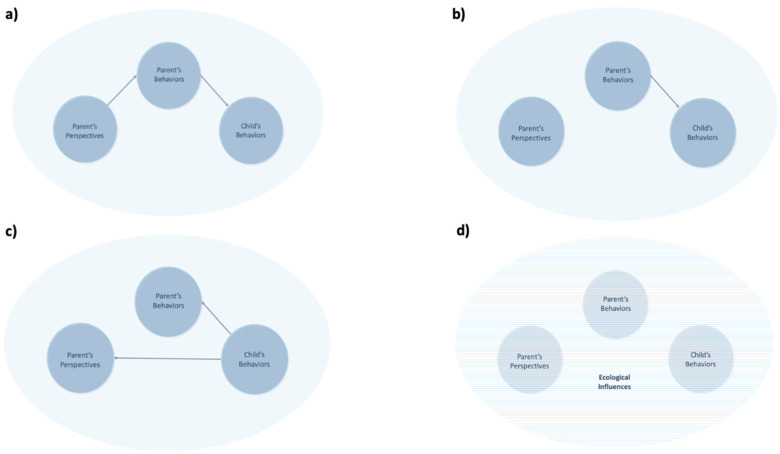
Components of the integrated model and ecological influences. *Note*: This figure series builds cumulatively to depict the integrated model. Each subfigure (**a**–**d**) introduces a distinct element, clarifying how parental perspectives, behaviors, child behaviors, and ecological context interact progressively. (**a**) shows how parental perspectives shape parental behaviors and indirectly influence children’s behaviors. (**b**) reveals the direct impact of parental behaviors on children’s motor development not directed by their perspectives. (**c**) focuses on children’s behaviors, emphasizing the reciprocal nature of the parent–child relationship, where children’s actions can influence parental behaviors and perspectives. (**d**) introduces the ecological layer, which is unique to each parent–child dyad, illustrating how these broader environmental contexts affect them.

At the heart of this model are parental perspectives—a wide range of cognitive constructs that parents hold regarding their children, themselves, the course of development, and child-rearing practices. These perspectives encompass knowledge, beliefs, attitudes, theories, and expectations. The existing research has explored aspects of parental perspectives under the term “parental cognitions”, which refers to parenting knowledge, beliefs, expectations, perceptions, and satisfaction [5,83]. Parents acquire information about child development through their daily experiences, such as babysitting siblings; through communicating with others, such as friends and family; and through education, such as parenting classes. For example, when a father sees his daughter turning her head side-to-side, he may know that she is hungry because he learned this from a parenting book. Additionally, parents form beliefs, theories, and ethnotheories [84] based on their own experiences, cultural backgrounds, and interactions with their children. For instance, a parent who experienced physical play during childhood may believe that physical play is essential for child development. Parents also develop expectations regarding their children’s developmental milestones. For example, a mother may expect her daughter to start walking around 12 months based on observations of other children. These perspectives guide parental behaviors, which, in turn, influence children’s behaviors (see Figure 2a and Figure 3a). Our model builds on the previous literature by unifying these constructs under the umbrella of parental perspectives to better capture their interaction with infant development and environmental contexts.

Parental behaviors refer to the actions, responses, and methods parents use in child-rearing. These behaviors are shaped by parents’ knowledge, theories, attitudes, and beliefs [85]. For example, when a father sees his hungry daughter turning her head, he draws from his knowledge and responds by feeding her. A mother who believes motor stimulation is beneficial might reinforce tummy time with her son. Parents’ expectations also influence how they interact with their children. For example, a mother who expects her child to walk early may provide more opportunities to practice walking. It is also important to note that parental perspectives do not always align with actual practices. A father may believe screen time is harmful for motor development while allowing his child to watch TV due to exhaustion. Nevertheless, parental behaviors play a crucial role in shaping children’s motor development (see Figure 3b).

Children’s behaviors in this model refer to the observable motor actions that infants display through their interactions with caregivers and their environment. These behaviors include both immediate reactions to parental actions and the motor skills that develop over time. For instance, children whose parents encourage sitting may acquire sitting skills faster, and those whose parents restrict walking may develop walking skills slower. Due to the reciprocal nature of parent–child dynamics, children’s behaviors can also influence their parents’ behaviors and perspectives (see Figure 2b and Figure 3c). While traditional models have viewed the child as a passive recipient of parental guidance, this model aligns with the concept of bidirectional influences between parents and children, as described by Relation Developmental Systems-Based models [86]. Our model also draws from Piaget’s and Gibson’s notion of children as active learners. Ref. [87] suggested that infants explore and experiment with motor actions to construct knowledge about their abilities and everything around them. Similarly, ref. [88]’s theory of perception emphasized that children are attuned to environmental cues, learning from the opportunities their surroundings provide. Together, these works indicate that children actively shape their own motor development through exploration, creating a feedback loop where their actions elicit meaningful responses from their caregivers. This bidirectional process also reflects the concept of developmental cascades, where a child’s motor development sparks environmental responses, further shaping growth [30]. For example, if an infant does well during tummy time, the parent may continue the activity, which may alter the parent’s perception of their child’s abilities.

Parents and children alone do not fully encapsulate the complexity of child development. Integrating an ecological layer is crucial to account for the additional factors that affect this process. Drawing from Bronfenbrenner’s ecological systems theory [45] and its revised version [48], we propose that parental perspectives, parental behaviors, and children’s behaviors are embedded within an ecological layer (see Figure 2c,d and Figure 3d). This layer includes, but is not limited to, factors such as neighborhoods, extended families, community networks, social trends, cultural contexts, public policies, socioeconomic status, and life events. More importantly, contemporary ecological factors, such as increased screen time, dual-working parents, shifting parenting norms, and access to developmental information through digital platforms, also shape parents, making them important contextual variables in modern motor development. These factors are incorporated into a flexible and inclusive ecological layer, acknowledging that not all families engage with every factor in the same way. These ecological factors influence parenting through a variety of mechanisms. For example, cultural norms shape what behaviors are encouraged or discouraged (e.g., prioritizing walking vs. crawling), while education systems and access to information affect parents’ knowledge about developmental milestones. Social support networks—including extended family or community groups—can provide reinforcement or challenges to parents’ child-rearing practices. Socioeconomic conditions may limit or expand the physical space, time, or safety available for motor play. Even national health policies or campaigns (such as supine sleep recommendations) can unintentionally alter daily caregiving routines. These contextual forces do not operate in isolation; they often interact with each other and with parents’ own experiences to shape the perspectives they hold and the behaviors they enact. Thus, the ecological layer in our model functions not only as a backdrop, but as a dynamic set of influences that help explain the variability in parental decision-making across cultural and socioeconomic contexts.

The relationships among parental perspectives, parental behaviors, and children’s motor development are conceptualized as correlational, dynamic, and bidirectional. Rather than assuming fixed causal pathways, our model highlights how these components mutually influence and reflect one another over time. These patterns unfold within broader ecological contexts, which shape and are shaped by the parent–child dyad. We do not claim that any of the model components directly determine motor outcomes. Instead, we propose that they are associated with and play an important part in infant motor development. This framework is testable using correlational and longitudinal designs, such as studies examining whether specific parental perspectives are associated with variations in motor milestones over time. While not prescriptive or predictive, the model offers a structure for generating empirically grounded questions about how parent and child factors interact within complex ecological systems. A comparative table of the proposed model and previous models can be found below (see Table 1).

## 5. Discussion

The proposed integrated model for understanding infant motor development provides a comprehensive framework that addresses parental perspectives, parental behaviors, and child behaviors within an ecological context. By emphasizing the evolving, reciprocal interactions among these components, this model offers a holistic perspective on the factors that influence motor development during infancy.

### 5.1. Summary of Proposed Model

Central to this model are parental perspectives, which play a key role in shaping how parents interact with their children and the behaviors they adopt. These perspectives also determine the opportunities and support children receive for motor development. Importantly, parental perspectives are not fixed; they change as parents gain new experiences, insights, and as they respond to their children’s behaviors. These shifting perspectives, in turn, guide their actions and practices.

Equally important are children’s behaviors, which encompass the observable motor actions and reactions that emerge as they engage with their environment and caregivers. These behaviors are influenced by parental perspectives and actions but also affect how parents respond. This aligns with developmental theories that have pointed out the active role children play in shaping their own development [87,88]. Rather than viewing parent–child interactions as a one-way process, this model highlights the cyclical nature of these exchanges, where children’s actions continuously inform parental behaviors and beliefs [86].

The proposed model is also unique as it places parents in the inner circle, examining the factors influencing their perspectives and behaviors. Such an approach not only highlights the critical role parents play during infancy but also provides a deeper understanding of why parents engage in specific parenting behaviors. This understanding can inform targeted interventions, enabling us to better help parents so that they, in turn, can better support their children.

The ecological layer integrates various contextual factors into a flexible framework, recognizing that each family operates within a unique set of circumstances. As societal conditions evolve, so too do the environments that influence motor development. By incorporating elements of prior theories, this model underlines the changing nature of children’s developmental contexts, while accounting for the distinct challenges and opportunities that families encounter [45,48]. Ultimately, the interactions among parents, children, and their surrounding environment jointly craft parenting practices and developmental outcomes.

### 5.2. Implications for Research and Practice

The integrated model has significant implications for both research and practice. Researchers can use this model to design studies that capture the complexity of environmental influences on motor development, employing methodologies that account for the interactions among different contextual factors. Observing and documenting changes in motor behaviors within various contexts will provide valuable insights into the developmental process and offer a clearer understanding of motor skill acquisition.

For practitioners, this model can guide the development of interventions that address multiple layers of influence, from direct parent–child interactions to broader community and societal supports. By focusing on enhancing parental perspectives and behaviors, interventions can create supportive environments that foster motor development. Understanding the unique circumstances of each family can also inform culturally sensitive and contextually relevant approaches.

### 5.3. Limitations and Future Directions

Despite its contributions, the model has a few limitations. It is based on correlational patterns and does not establish causal mechanisms. While this reflects the complexity of real-world development, it also limits its predictive precision. Additionally, the model does not explicitly account for factors, such as parental mental health, child temperament, and child genetic/innate processes, that may moderate or mediate parent–child dynamics and influence child motor outcomes. Its applicability to high-adversity contexts—such as children who are cared for by caregivers other than their parents—may be limited. Further, while it centers on parental perspectives and behaviors, it does not yet incorporate children’s own beliefs, which may be relevant in older age groups and should be addressed.

The primary focus of the current review and proposed model is on motor development during infancy. However, the proposed model does not need to be limited to the motor domain. Examining the applicability of our proposed model to other developmental domains would be beneficial and should be entertained in future research. Further, longitudinal studies examining how parental perspectives evolve over time and how these changes correlate with children’s motor trajectories would enhance our understanding of the reciprocal nature of child–parent interactions over time.

Finally, another important direction for future work involves understanding where parents’ perspectives come from. One source is formal parenting education, which varies across countries and systems. A recent analysis by Lobo et al. [89] compared publicly available parent education materials and found that motor development was discussed quite often, yet the content varied by country. Future research should explore how their content and other parenting resources influence caregivers and, in turn, infants’ motor behaviors.

## 6. Conclusions

Parental perspectives and behaviors shape and influence a child’s development across domains. However, research on the impact of parental perspectives or behaviors on infant motor development remains rare. This may be due to the lack of a theoretical model that focuses on how parental perspectives and behaviors within a specific ecological context act to shape infant motor behaviors and development specifically. To address this gap, we propose a new holistic model centered on infants’ observable motor behaviors and explicitly include parental perspectives and behaviors as factors shaping motor developmental outcomes. In contrast to prior models, the proposed model separates parental beliefs from their behaviors, situates parent–child interactions within a larger ecological context, and includes bidirectional relations between infant behavior and both parental beliefs and behaviors. We hope that the proposed model will be applied to prior research and will generate new hypotheses for future research on the complex factors shaping infant motor development.

## Figures and Tables

**Table 1:** Comparison of our model with previous models.

Feature	Maturational Theory	Dynamic Systems Theory	Sociocultural Theory	Ecological Systems Theory	Proposed Integrated Model
Central Focus	Biological maturation of the child	Interplay of internal and external systems	Social interaction and guided learning	Multilayered environmental influences on the child	Parent-child dyad within ecological context
View of Parent	Peripheral, minor environmental influence	Implicit part of the system	“More knowledgeable other”, but secondary to child	One of many microsystem agents	Active, reflective agent influencing development
Mechanism of Change	Genetically driven maturation	Self-organization and adaptation	Scaffolding within ZPD	Systemic and bidirectional influences	Reciprocal, evolving influence situated in the context
Parental Perspectives Addressed	No	No	Limited	Indirectly	Central and directly defined
Applicability to Motor Development	Yes, but limited to biological factors	Yes, but concept is too abstract	Not motor-specific	Not motor-specific	Specifically designed for infant motor development
Bidirectional Influence	No	Yes	Yes	Yes	Yes
Accounting for Variability in Developmental Context	Assumes universal biological sequence; minimal contextual sensitivity	Acknowledges context, but lacks specificity in application	Cultural learning varies, but often child-centered	Includes multiple systems, but model is generalized across groups	Context-sensitive and individualized; adapts to each family’s unique ecological environment

Note: This table compares the key characteristics of the major developmental theories—maturational theory, dynamic systems theory, sociocultural theory, ecological systems theory—with the proposed model. It highlights the distinctive features of each framework across several dimensions, including their focus, treatment of parental roles, mechanisms of change, inclusion of parental perspectives, applicability to motor development, model direction, and considerations of varied developmental contexts.

## Data Availability

No new data were created or analyzed in this study.
